# WWOX binds MERIT40 and modulates its function in homologous recombination, implications in breast cancer

**DOI:** 10.1038/s41417-023-00626-x

**Published:** 2023-05-29

**Authors:** Karim Taouis, Sophie Vacher, Josée Guirouilh-Barbat, Jacques Camonis, Etienne Formstecher, Tatiana Popova, Anne-Sophie Hamy, Ambre Petitalot, Rosette Lidereau, Sandrine M. Caputo, Sophie Zinn-Justin, Ivan Bièche, Keltouma Driouch, François Lallemand

**Affiliations:** 1 Service de génétique, unité de pharmacogénomique, Institut Curie, 26 rue d’Ulm, Paris, France; 2 Paris Sciences Lettres Research University, Paris, France; 3 Laboratoire Recombinaison-Réparation et Cancer UMR8200 Stabilité Génétique et Oncogenèse Institut Gustave Roussy, PR2, pièce 426114 Rue Edouard Vaillant, 94805 Villejuif, France; 4 INSERM U528, Institut Curie, 26 rue d’Ulm, Paris, France; 5 Hybrigenics Services, 1 rue Pierre Fontaine, 91000 Evry, France; 6 Centre De Recherche, Institut Curie, Paris, F-75248 France; 7 INSERM U830, Paris, F-75248 France; 8 Residual Tumor & Response to Treatment Laboratory, RT2Lab, Translational Research Department, INSERM, U932 Immunity and Cancer, University Paris, Paris, France; 9 Department of Medical Oncology, Institut Curie, Paris, France; 10 University Paris, Paris, France; 11 Institute for Integrative Biology of the Cell, CEA, CNRS, Université Paris-Sud, Gif-sur-Yvette, France; 12 INSERM U1016, Université Paris Descartes, 4 avenue de l’observatoire, Paris, France

**Keywords:** Breast cancer, Molecular biology

## Abstract

The tumor suppressor gene *WWOX* is localized in an unstable chromosomal region and its expression is decreased or absent in several types of cancer. A low expression of *WWOX* is associated with a poor prognosis in breast cancer (BC). It has recently been shown that WWOX contributes to genome stability through its role in the DNA damage response (DDR). In breast cancer cells, WWOX inhibits homologous recombination (HR), and thus promotes the repair of DNA double-stranded breaks (DSBs) by non-homologous end joining (NHEJ). The fine-tuning modulation of HR activity is crucial. Its under or overstimulation inducing genome alterations that can induce cancer. MERIT40 is a positive regulator of the DDR. This protein is indispensable for the function of the multi-protein complex BRCA1-A, which suppresses excessive HR activity. MERIT40 also recruits Tankyrase, a positive regulator of HR, to the DSBs to stimulate DNA repair. Here, we identified MERIT40 as a new molecular partner of WWOX. We demonstrated that WWOX inhibited excessive HR activity induced by overexpression of MERIT40. We showed that WWOX impaired the MERIT40-Tankyrase interaction preventing the role of the complex on DSBs. Furthermore, we found that *MERIT40* is overexpressed in BC and that this overexpression is associated to a poor prognosis. These results strongly suggest that WWOX, through its interaction with MERIT40, prevents the deleterious impact of excessive HR on BC development by inhibiting MERIT40-Tankyrase association. This inhibitory effect of WWOX would oppose MERIT40-dependent BC development.

## Introduction

Genome integrity is essential for cell viability and prevents genetic alterations that can cause cancer [1–3]. Cells have developed several DNA repair mechanisms and cell cycle checkpoints that ensure repair of DNA damages before cell cycle progression resumes. One of the most severe forms of DNA damage which can result in chromosomal aberrations, is DNA double-strand break (DSB) [1]. Two major molecular mechanisms are involved in the DSBs repair: Homologous Recombination (HR) and Non Homologous End Joining (NHEJ) [4]. In HR, recombination occurs between sister chromatids present during the S and G2 phases of the cell cycle. HR activity has to be finely regulated in cells, because too low or too high HR activity induces genome instability [5–10]. NHEJ is active throughout the cell cycle, and joins broken ends by simple religation.

The repair of DSBs is induced by the recruitment of various protein complexes at the DNA breaks which results in the formation of nuclear foci [11]. The MRN complex formed by MRE11, RAD50 and NBS1, recognizes and binds the DSBs. Then, it recruits and activates ATM, which induces HR or NHEJ depending on the intracellular context and DSBs localization [12]. To activate HR, ATM phosphorylates different substrates such as BRCA1, encoded by the breast cancer susceptibility gene *BRCA1* [13]. BRCA1 is involved in three complexes playing critical roles in HR: BRCA1-A, BRCA1-B and BRCA1-C [14]. BRCA1-B, composed of BRCA1, Brip1/BACH1 and TopB1, and BRCA1-C, comprised of BRCA1, CtIP, and MRN, promote resection of the DNA ends on either side of the DSBs to generate single stranded ends, which are then coated sequentially with RPA and RAD51. The resulting nucleoprotein filaments invade the complementary strand of the sister chromatid, allowing DNA synthesis to take place.

MERIT40 (Mediator of RAP80 Interaction and Targeting 40 kDa protein) plays an important role in DNA Damage Response (DDR). Indeed, the depletion of MERIT40 impairs G2/M checkpoint control and reduces cell survival following irradiation (IR) treatment [15–19]. In response to DNA damages induced by IR, MERIT40 accumulates to DNA damage sites thus forming nuclear foci [15–17]. This protein is a member of the nuclear complex BRCA1-A also composed of ABRAXAS, RAP80, BRE, and BRCC36. MERIT40 is essential for the formation of this complex. BRCA1-A eliminates excess HR to prevent genome instability which can cause cancer, by limiting the extend of DNA end resection [7, 20]. Additionally, MERIT40 recruits the poly (ADP-ribose) polymerase Tankyrase, a positive regulator of HR, to DSBs to stimulate DNA repair, strongly suggesting that MERIT40 could stimulate HR by recruiting Tankyrase to DSBs [18, 21].

*WWOX* gene (WW domain-containing- oxydoreductase) located on the common fragile site FRA16D on chromosome 16q23.2, encodes a protein composed of two N-terminal WW domains (WW1 and WW2) and one C-terminal short-chain dehydrogenase/reductase domain (SDR) [22–24]. WW domains are responsable for the interaction of WWOX with a large set of protein partners involved in several cancer-related molecular pathways [25]. WWOX expression is inhibited or absent in many cancers such as BC. Various animal models have highlighted a tumor suppressive activity of WWOX in BC. However, the molecular mechanism by which WWOX performs its anti-tumor activity is still a matter of debate. Recently, it has been demonstrated that WWOX is involved in maintaining genome integrity by modulating different DNA damage repair mechanisms [26–28]. WWOX inhibits HR thus promoting the DSBs repair by NHEJ in human breast cancer cells [28]. WWOX inhibits HR by preventing the formation of BRCA1-B and BRCA1-C complexes and therefore resection. Inhibition of the formation of these complexes by WWOX depends on its ability to interact with BRCA1 [29].

In this article, we identified MERIT40 as a new molecular partner of WWOX. This finding prompted us to study the role of the WWOX-MERIT40 interaction in HR in the context of BC.

## Results

### WWOX interacts with MERIT40

In order to better define the molecular function of WWOX, we performed a yeast two-hybrid screen to identify new molecular partners of this protein [30–32]. As baits, we used a WWOX isoform (WWOX variant 2, WWOXv2, NP_570607) containing only the WW1 and WW2 domains, and a truncated SDR domain. We screened at saturation a highly complex human placenta library, the N-terminus region of MERIT40 (amino acid sequence 43–206) were found to interact with the bait. MERIT40 was recovered as a prey in only 2 screens in more than 2.300 performed on the same library strongly suggesting that the WWOX-MERIT40 association is highly specific (E. Formstecher personal communications). Moreover, because this interaction was detected in yeast, it is very likely that it is a direct interaction (E. Formstecher personal communications [33]).

**Fig. 1:**
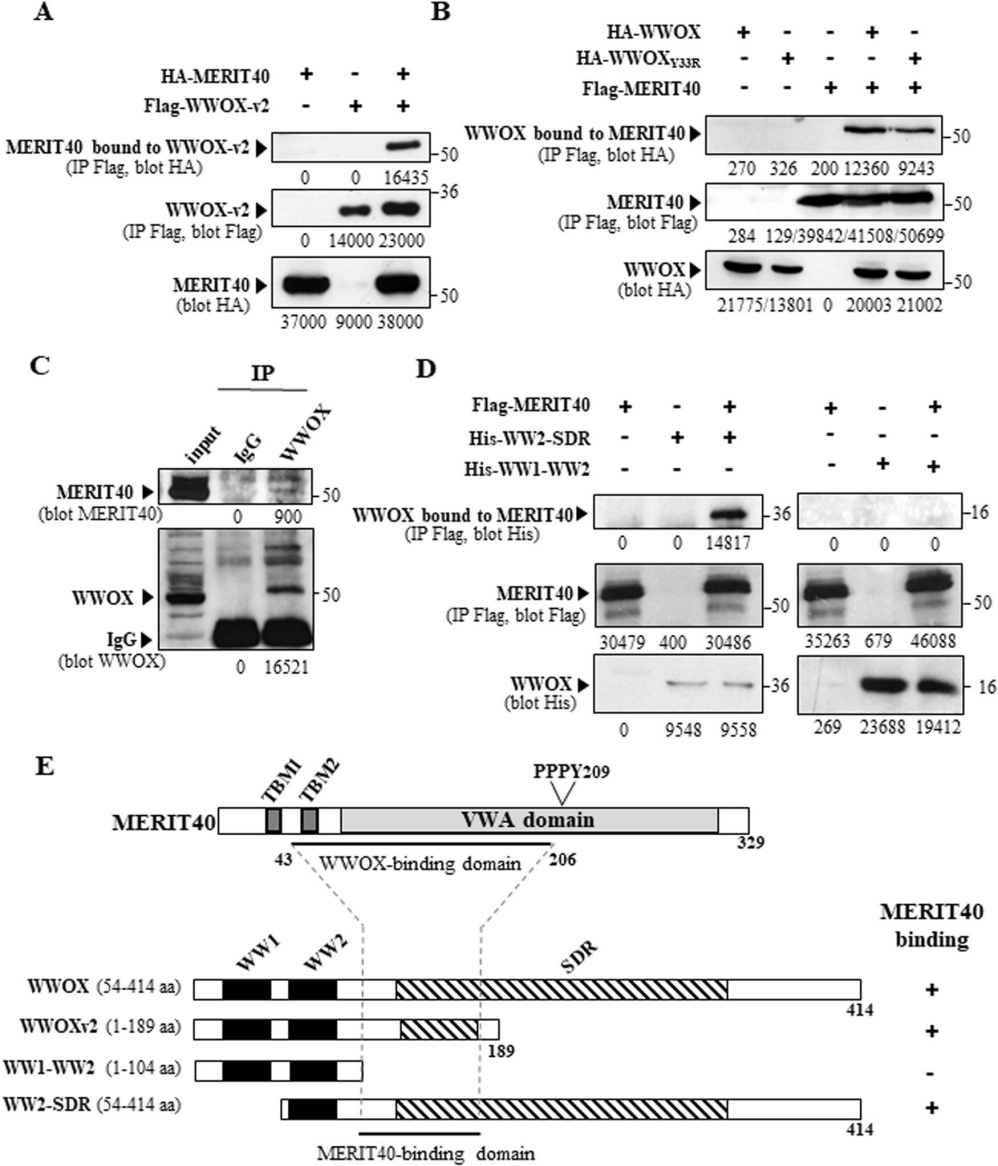
WWOX-MERIT40 interaction. **A** WWOXv2-MERIT40 interaction. HEK293 cells transfected with Flag-WWOXv2 and HA-MERIT40 as indicated were immunoprecipitated with anti-Flag antibody. Immunoprecipitates and cell lysates were examined by immunoblotting with anti-HA and anti-Flag antibodies. **B** WWOX and WWOX_Y33R_ interact with MERIT40 in vivo. HEK293 cells transfected with HA-WWOX, HA-WWOX_Y33R_, and Flag-MERIT40 as indicated were immunoprecipitated with anti-Flag antibody (IP Flag). Immunoprecipitates and cell lysates were examined by immunoblotting with anti-HA (blot HA) and anti-Flag (blot Flag) antibodies. **C** WWOX and MERIT40 form an endogenous complex in the BC cells MCF7. MCF7 cell extract was immunoprecipitated with anti-WWOX antibody; immunoprecipitate and cellular extracts were examined by immunoblotting with anti-MERIT40 and anti-WWOX antibodies as indicated. **D** Binding domains of WWOX for MERIT40. HEK293 transfected with His-WW1-WW2, His-WW2-SDR, and Flag-MERIT40 as indicated were immunoprecipitated with anti-Flag antibody. Immunoprecipitates and cell lysates were examined by immunoblotting with anti-His and anti-Flag antibodies as indicated. **E** Schematic representation of MERIT40, the WWOX-binding domain of MERIT40 identified by yeast two-hybrid and the MERIT40-binding domain are indicated by a black lines. TMB: Tankyrase-binding motif. VWA domain: Von-Willebrand factor A domain.

To validate the WWOX-MERIT40 association, we performed transient transfections and co-immunoprecipitation experiments in HEK293 cells. We were able to visualize WWOXv2-MERIT40 and WWOX-MERIT40 interactions in these cells (Fig. 1A, B). To detect the WWOX-MERIT40 complex at the endogenous level in BC cells, we first determined the expression of MERIT40 in several human BC cell lines. We found a strong expression of this protein in all examined breast cell lines (Fig. S1). We used MCF7 cells expressing high levels of both WWOX and MERIT40 to perform our co-immunoprecipitation assays. We found that WWOX co-immunoprecipitated with MERIT40 indicating that endogenous WWOX and MERIT40 proteins interact with each other in BC cells (Fig. 1C).

The WW1 domain of WWOX mediates interactions with numerous proteins through their PPXY consensus motifs [34]. MERIT40 possesses a PPXY motif: PPPY209. However, surprisingly, this motif is located in the VWA domain of MERIT40 which is mostly buried in BRCA1-A complex [35]. Moreover, this motif is not present in the region interacting with WWOX as determined by the two-hybrid screen (amino acid 43–206). Therefore, we hypothesized that the WW1 domain of WWOX is not involved in the WWOX-MERIT40 interaction. To validate this hypothesis, we performed co-immunoprecipitation assays with a mutant form of WWOX harboring a Y33R point mutation in the WW1 domain inhibiting its ability to interact with the PPXY motifs of different proteins [32, 36–38]. We performed co-immunoprecipitation assays to reveal the interaction of WWOX and WWOX_Y33R_ with MERIT40. Consistent with our hypothesis, we showed that this mutation did not affect the WWOX-MERIT40 association (Fig. 1B). We also examined the ability of different WWOX domains to associate with MERIT40. We found that the N-terminal region of WWOX containing only the WW1 and WW2 domains did not interact with MERIT40, whereas the region composed of the WW2 and SDR domains did (Fig. 1D).

Altogether, these results define MERIT40 as a new molecular partner of WWOX and suggest that the domain of WWOX interacting with MERIT40 is located in the N terminal part of the SDR domain (Fig. 1E).

### WWOX inhibits MERIT40-induced HR

Several studies strongly suggest that MERIT40 is a positive regulator of HR [10–12, 14, 19–21, 37]. Therefore, we investigated the effect of WWOX on MERIT40’s ability to modulate this DNA repair mechanism. We used the DR-GFP assay (Direct Repeat-Green Fluorescent Protein assay), in which reconstitution of a defective GFP gene in Hela cells (DR-GFP Hela) depends on HR-mediated repair [39]. We quantified HR activity as the fraction of GFP-positive cells.

**Fig. 2:**
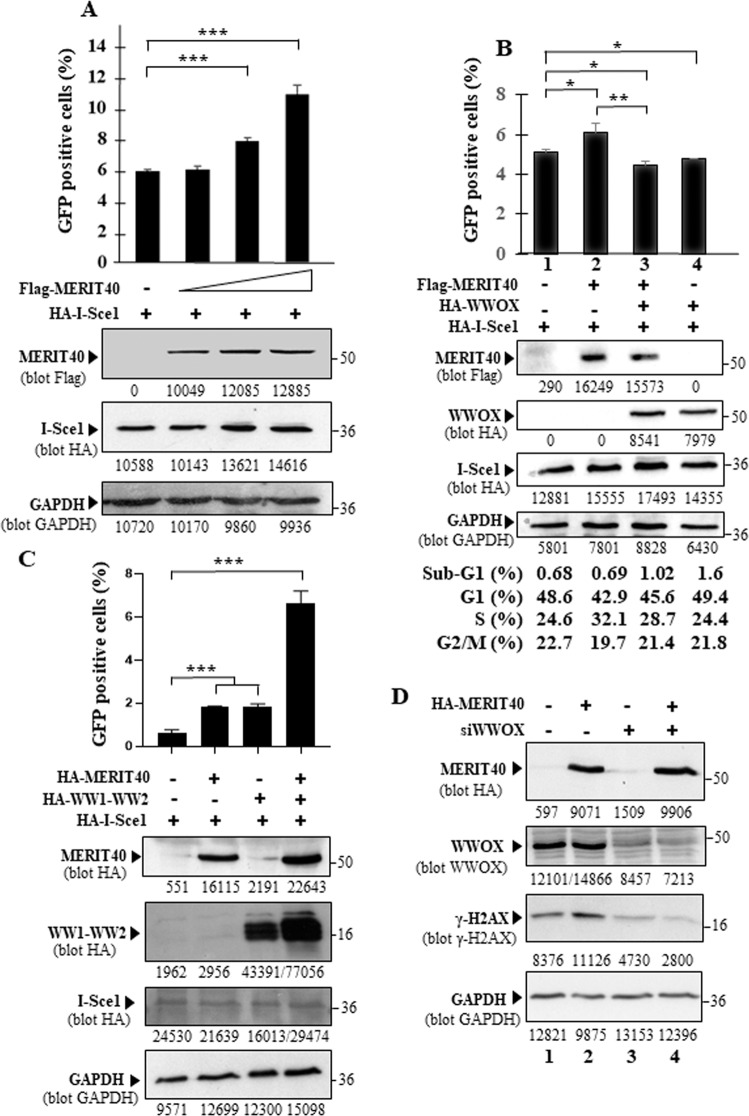
WWOX counteracts the overstimulation of HR induced by MERIT40. **A** Effect of MERIT40 on HR-mediated repair of I-Sce1 induced DSBs in Hela cells. DR-GFP Hela cells were transfected with HA-I-Sce1 and Flag-MERIT40 as indicated. After 48 h, the number of GFP-positive cells was determined by flow cytometry. The western blots show the expressions of HA-I-Sce1, GAPDH (loading control), and Flag-MERIT40. **B** Effect of WWOX on the MERIT40’s ability to overstimulate HR. DR-GFP-Hela cells were transfected with HA-I-Sce1, Flag-MERIT40, and Myc-WWOX as indicated. After 48 h, the number of GFP-positive cells for each experimental condition were determined by flow cytometry. The western blots show the expressions of HA-I-Sce1, GAPDH, Flag-MERIT40, and Myc-WWOX. For each experimental condition, the repartition of cells in the different phases of the cell cycle is determined by flow cytometry analysis. **C** Effect of a truncated form of WWOX composed only of its WW1 and WW2 domains (WW1–WW2) on the MERIT40’s ability to overstimulate HR. DR-GFP Hela cells were transfected with HA-I-Sce1, HA-WW1-WW2 and HA-MERIT40 as indicated. After 48 h, the number of GFP-positive cells was determined by flow cytometry. The western blots show the expressions of HA-I-Sce1, GAPDH, HA-WW1-WW2 and HA-MERIT40. **A**–**C** Experiments were performed in triplicate, and error bars indicate standard deviation. **D** Effect of ectopic expression of MERIT40 and inhibition of WWOX expression on expression of the DNA damage marker γ–H2AX. HEK293 were transfected with siRNA control (lanes 1 and 2) or siRNA WWOX (siWWOX, lanes 3 and 4). After 72 h, cells were transfected once again with the siRNA. After 24 h, cells were transfected with HA-MERIT40 as indicated. After 48 h, cells were treated with cisplatin (3 µg/ml) for 1 h and allowed to recover for 6 h, cells were then lysed. Cell lysates were examined by immunoblotting with anti-HA, anti-WWOX, and anti-GAPDH and anti-γ-H2AX antibodies.

Transient transfection of DR-GFP Hela cells with the endonuclease I-Sce1 alone or with MERIT40 allowed us to show that cells overexpressing MERIT40 and I-Sce1 exhibited higher HR amplitude compared to cells expressing only I-Sce1, indicating that overexpression of MERIT40 induces excessive HR activity (Fig. 2A). In agreement with the results of Schrok et al. [28], we observed that ectopic expression of WWOX inhibited HR. We also observed that this ectopic expression inhibited excessive HR activity induced by MERIT40 (Fig. 2B). By performing flow cytometry analyses, we found that WWOX did not induce neither apoptosis (evaluated by the percentage of cells in sub-G1) nor cell cycle arrest in our experimental conditions (Fig. 2B). We confirmed that WWOX did not impact cell death by showing that I-Sce1 expression was similar in cells transfected or not with WWOX (Fig. 2B compare lane 1 to lane 4 of the third panel from the top). We found that ectopic expression of a truncated form of WWOX composed only of its WW1 and WW2 domains, strongly increased HR and the positive effect of MERIT40 on HR, suggesting that this truncated form of WWOX acts as a dominant negative suppressing the negative effect of WWOX on HR (Fig. 2C). We also found that inhibition of WWOX expression inhibited the expression of the molecular marker of DNA damage γ-H2AX, this effect of WWOX was strongly enhanced in presence ectopic expression of MERIT40. These results strongly suggest that WWOX inhibition or MERIT40 activation induces an important DNA damage repair activity (Fig. 2D).

Altogether, these results indicate that WWOX is able to inhibit the positive effect of overexpression of MERIT40 on HR without modulating apoptosis or cell progression through the cell cycle.

### WWOX does not prevent the formation of IR-induced MERIT40 nuclear foci

Accumulation of MERIT40 at DSBs is crucial for repair of DNA damages induced by IR [15–19]. To determine the mechanism by which WWOX inhibits MERIT40-induced HR, we therefore assessed the effect of WWOX on the ability of MERIT40 to form nuclear foci after IR treatment.

Hela cells were transfected with MERIT40 or both MERIT40 and WWOX expression vectors. Then, cells were irradiated with 10 Gy and allowed to recover before immunostaining.

**Fig. 3:**
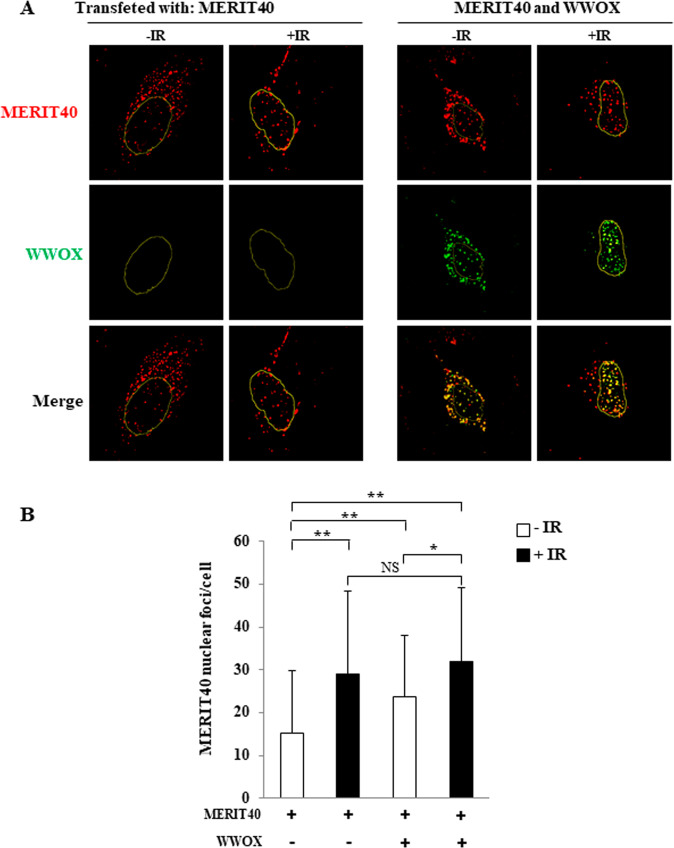
Effect of WWOX on the ability of MERIT40 to form nuclear foci. **A** Hela cells were transfected with MERIT40 and WWOX expression vectors as indicated. After 48 h, cells were irradiated with 10 Gy and allowed to recover for 4 h before permeabilization, fixation, and immunostaining with anti-MERTI40 (red) and anti-WWOX (green) antibodies. The contours of the nuclei are indicated. **B** Histogram is the quantitation of the results (50 cells were counted in each condition, the experiment was done once).

We observed that IR increased the number of ectopic MERIT40 nuclear foci in Hela cells, indicating that these foci corresponded to MERIT40 located at DNA damages sites (Fig. 3A). Ectopic WWOX colocalized with ectopic MERIT40 both in the cytoplasm and nucleus, in agreement with the ability of WWOX and MERIT40 to interact. Most importantly, WWOX did not decrease the number of MERIT40 nuclear foci in irradiated cells. Interestingly, WWOX significantly increased the number of MERIT40 nuclear foci in non-irradiated cells (Fig. 3B).

Therefore, WWOX does not inhibit the ability of MERIT40 to activate HR by preventing its capacity to form nuclear foci in response to DSBs.

### WWOX inhibits the MERIT40-Tankyrase interaction

It has previously been demonstrated that MERIT40 recruits Tankyrase, a positive regulator of HR, to DSBs to induce the DDR following IR treatment [18, 21]. We confirmed that MERIT40 is an activator of HR (Fig. 2B, C). We therefore examined the possibility that WWOX inhibits the effect of MERIT40 on HR by preventing MERIT40-Tankyrase association.

**Fig. 4:**
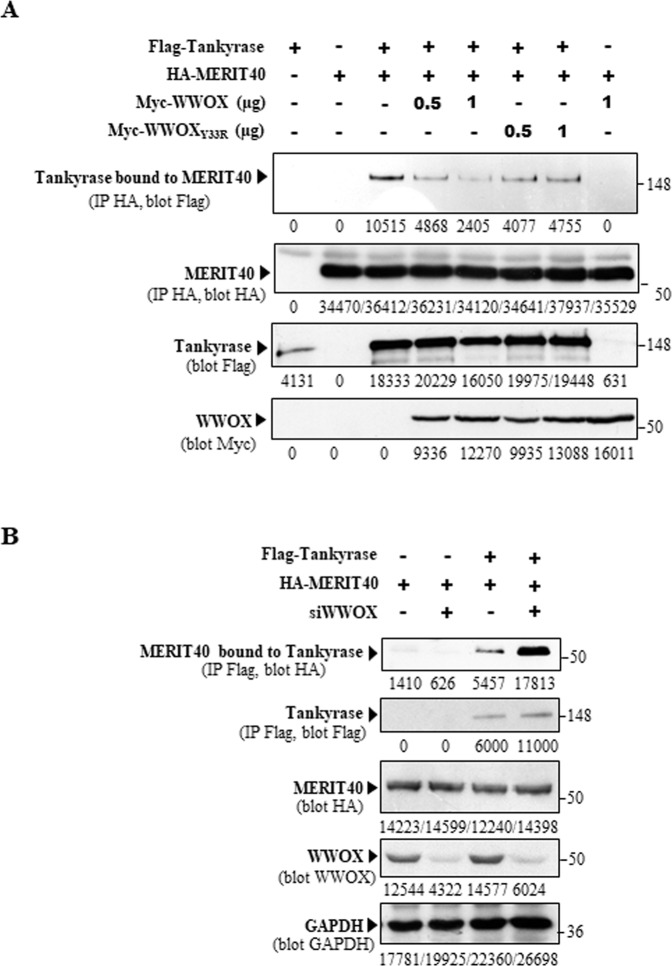
WWOX inhibits the MERIT40-Tankyrase association. **A** Ectopic expression of WWOX inhibits the MERIT40-Tankyrase association. HEK293 transfected with Myc-WWOX, Myc-WWOX_Y33R_, HA-MERIT40, and Flag-Tankyrase as indicated, were immunoprecipitated with anti-HA antibody. Immunoprecipitates and cell lysates were then examined by immunoblotting with anti-HA, and anti-Myc, and anti-Flag antibodies. **B** Inhibition of endogenous WWOX expression stimulates MERIT40-Tankyrase association. HEK293 were transfected with siRNA control (lanes 1 and 3) or siRNA WWOX (lanes 2 and 4). After 72 h, cells were transfected once again with the siRNA. After 24 h, cells were transfected with HA-MERIT40 and Flag-Tankyrase as indicated. After 48 h, cells were immunoprecipitated with anti-Flag antibody. Immunoprecipitates and cell lysates were then examined by immunoblotting with anti-HA, anti-Flag, anti-WWOX, and anti-GAPDH antibodies.

MERIT40 contains two Tankyrase-binding motifs (TBM1 and TBM2) required for MERIT40-Tankyrase association [18]. TBM2 is located within the WWOX-binding domain determined by our two-hybrid screen (Fig. 1E) supporting the hypothesis that WWOX may compete with Tankyrase for binding to MERIT40. We thus, assessed whether WWOX expression could alter MERIT40-Tankyrase association. We found that MERIT40 highly increased the stability of Tankyrase as previously described (Fig. 4A compare lane 1 to lane 3 of the third panel from the top) [19]. Most importantly, ectopic expression of WWOX as well as that of WWOX_Y33R_, inhibited the MERIT40-Tankyrase interaction (Fig. 4A). Conversely, we demonstrated that inhibition of endogenous WWOX expression upon specific WWOX siRNA treatment enhances this interaction (Fig. 4B).

Altogether, our results suggest that WWOX inhibits the ability of MERIT40 to induce HR by binding to its TBM2 motif and thus preventing the MERIT40-Tankyrase interaction.

### High expression of *MERIT40* is a marker of poor prognosis in BC

We showed that high expression of MERIT40 induces excessive HR activity (Fig. 2B, C). Given that overstimulation of HR causes genome instability which can induce cancer [1, 3, 8, 10], we reasoned that MERIT40 overexpression could promote the cancer progression.

To study this possibility, we analyzed the expression of *MERIT40* in 499 breast tumors (Curie cohort) classified into four subtypes according to their hormone receptors (ER/PR) and ERBB2 status (see Materials and Methods) (Table 1).

**Table 1:** Characteristics of the 499 breast tumors.

	Number of patients (%)	Number with metastases (%)	*p* value^a^
Total	499 (100)	199 (100)	
Age			
≤50	118 (23.6)	48 (40.7)	0.75 (NS)
>50	381 (76.4)	151 (39.6)	
SBR histological grade^b,c^			
I	58 (11.9)	11 (19.0)	**0.0015**
II	229 (47.0)	97 (42.4)	
III	200 (41.1)	88 (44.0)	
Lymph node status^d^			
0	152 (30.8)	46 (30.3)	<**0.0001**
1–3	239 (48.4)	85 (35.6)	
>3	103 (20.9)	66 (64.1)	
Macroscopic tumor size^e^			
≤25 mm	235 (48.0)	71 (30.2)	<**0.0001**
>25 mm	255 (52.0)	127 (49.8)	
ERα status			
Negative	168 (33.7)	70 (41.7)	0.14 (NS)
Positive	331 (66.3)	129 (39.0)	
PR status			
Negative	239 (47.9)	102 (42.7)	**0.046**
Positive	260 (52.1)	97 (37.3)	
ERBB2 status			
Negative	381 (76.4)	148 (38.8)	0.30 (NS)
Positive	118 (23.6)	51 (43.2)	
Molecular subtypes			
ER/PR− ERBB2−	96 (19.2)	35 (36.5)	**0.049**
ER/PR− ERBB2+	65 (13.0)	33 (50.8)	
ER/PR+ ERBB2−	285 (57.1)	113 (39.6)	
ER/PR+ ERBB2+	53 (10.6)	18 (34.0)	

*ER/PR* hormones receptors, *NS* not significant.

^a^Log-rank test.

^b^Scarff–Bloom Richardson, classification.

^c^Information available for 487 patients.

^d^Information available for 494 patients.

^e^Information available for 490 patients.

Significant values are shown in bold.

**Fig. 5:**
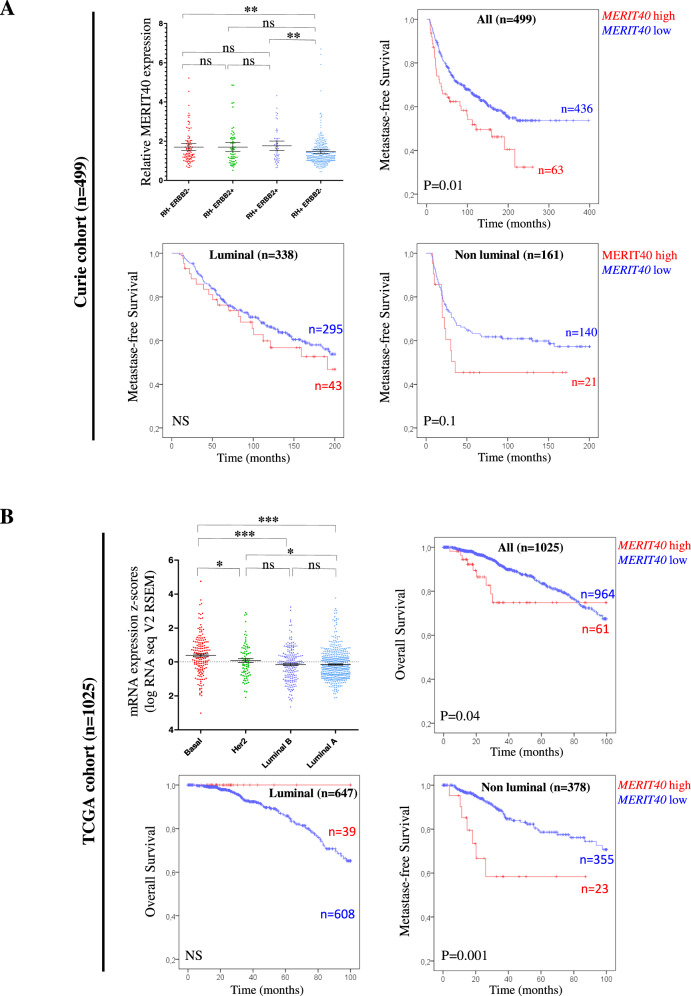
High expression of *MERIT40* is a marker of poor prognosis in BC. A real-time PCR analysis of *MERIT40* expression in the different breast cancer subtypes and Kaplan–Meier curves showing metastasis-free survival rates of patients with tumors expressing high (red line) vs. low (blue line) levels of *MERIT40* mRNA, in **A** Curie cohort (*n* = 499), and **B** TCGA cohort (*n* = 1025).

We first investigated *MERIT40* transcripts levels in this series of tumors as compared to normal breast tissue (*n* = 14) and found that *MERIT40* is overexpressed in breast tumors (*p* = 0.001; Fig. S2). Interestingly, higher levels of MERIT40 were observed in triple-negative breast cancers (ER/PR− ERBB2−) as compared to luminal tumors (ER/PR+ ERBB2−) (Fig. 5A).

We then examined the relationships between *MERIT40* expression (high *versus* low) and several classical clinicopathological parameters. We observed that high expression of *MERIT40* was associated with several markers of poor prognosis: SBR histological grade III (*p* = 0.03), high macroscopic tumor size (*p* = 0.0021), negative ERα status (*p* = 0.0052), negative PR status (*p* = 0.035), high *MKI67* mRNA expression (*p* < 0.0001), and molecular subtypes (*p* = 0.0021) (Table 2).

**Table 2:** Relationship between *MERIT40* transcripts levels and classical clinical biological parameters in a series of 499 breast cancers.

		Number of patients (%)		
	Total population (%)	Low *MERIT40* mRNA expression	High *MERIT40* mRNA expression	*p* value^a^
Total	499 (100)	436 (83.4)	63 (12.6)	
Age				
≤50	118 (23.6)	101 (23.2)	17 (27.0)	0.5 (NS)
>50	381 (76.4)	335 (76.8)	46 (73.0)	
SBR histological grade^b,c^				
I	58 (11.9)	53 (12.5)	5 (8.1)	**0.03**
II	229 (47.0)	207 (48.7)	22 (35.5)	
III	200 (41.1)	165 (38.8)	35 (56.5)	
Lymph node status^d^				
0	152 (30.8)	135 (31.2)	17 (27.9)	0.87 (NS)
1–3	239 (48.4)	208 (48.0)	31 (50.8)	
>3	103 (20.9)	90 (20.8)	13 (21.3)	
Macroscopic tumor size^e^				
≤25 mm	235 (48.0)	217 (50.6)	18 (29.5)	**0.0021**
>25 mm	255 (52.0)	212 (49.4)	43 (70.5)	
ERα status				
Negative	168 (33.7)	137 (31.4)	31 (49.2)	**0.0052**
Positive	331 (66.3)	299 (68.6)	32 (50.8)	
PR status				
Negative	239 (47.9)	201 (46.1)	38 (60.3)	**0.035**
Positive	260 (52.1)	235 (53.9)	25 (39.7)	
ERBB2 status				
Negative	381 (76.4)	341 (78.2)	40 (63.5)	**0.01**
Positive	118 (23.6)	95 (21.8)	23 (36.5)	
Molecular subtypes				
ER/PR− ERBB2−	96 (19.2)	78 (17.9)	18 (28.6)	**0.0021**
ER/PR− ERBB2+	65 (13.0)	53 (12.2)	12 (19.0)	
ER/PR+ ERBB2−	285 (57.1)	263 (60.3)	22 (34.9)	
ER/PR+ ERBB2+	53 (10.6)	42 (9.6)	11 (17.5)	
MKI67 mRNA expression^f,g^				
Median	12. 5 (0.80–313)	11.7 (0.80–313)	22.4 (2.69–117)	**<0.0001**

*ER/PR* hormone receptors, *NS* not significant.

^a^Chi-squared test.

^b^Scarff–Bloom Richardson classification.

^c^Information available for 487 patients.

^d^Information available for 494 patients.

^e^Information available for 490 patients.

^f^Information available for 429 patients.

^g^Mann–Whitney test.

Significant values are shown in bold.

We next performed a log-rank test to analyze the influence of *MERIT40* expression on metastasis-free survival (MFS). Patients with breast tumors expressing low levels of *MERIT40* had better MFS than patients with breast tumors expressing higher levels of this gene (*p* = 0.01) (Fig. 5A). Additionally, we evaluated the prognostic value of MERIT40 expression with regard to the different molecular subtypes of breast tumors. We thus determined that MERIT40 tended to be associated with a poor prognosis specifically in non-luminal tumors as compared to luminal subtype (*p* = 0.1; Fig. 5A).

Multivariate analysis using a Cox proportional hazards model assessed the predictive value for MFS of the parameters found to be significant in univariate analysis, i.e. SBR histological grade, lymph node status, macroscopic tumor size and PR status (Table 1), and *MERIT40* expression (*p* = 0.012) (Fig. 5A). The prognostic significance of lymph node status, macroscopic tumor size and *MERIT40* expression persisted in the multivariate analysis, indicating that *MERIT40* expression is an independent prognostic factor in BC (Table 3).

**Table 3:** Multivariate COX analysis of MFS for *MERIT40* expression levels in the series of 499 breast cancers.

Characteristics		HR^a^	95% CI^b^	*p* value^c^
*MERIT40*	≤2.205	1.0		**0.03**
	>2.205	1.53	1.04–2.25	
Macroscopic tumor size	≤25 mm	1.0		**0.0037**
	>25 mm	1.59	1.16–2.17	
Lymph node status	0	1.0		**<0.0001**
	1–3 and >3	1.64	1.33–2.03	
SBR histological grade	I	1.0		0.17 (NS)
	II and III	1.19	0.93–1.51	
PR status	Negative	1.0		0.27 (NS)
	Positive	0.84	0.61–1.15	

^a^Hazard ratio.

^b^95% confidential interval.

^c^Multivariate COX analysis.

Significant values are shown in bold.

To validate our findings, we assessed *MERIT40* expression and its prognostic value in an independent larger series of breast tumors. To do so, we analyzed the publically available expression data from the Cancer Genome Atlas (TCGA) -breast project (Fig. 5B). In agreement with our results on Curie cohort, *MERIT40* was significantly more highly expressed in the basal subtype of TCGA-breast tumors (Fig. 5B). Similarly, *MERIT40* was associated with a poor prognosis in the TCGA non-luminal tumors (*p* = 0.001, Fig. 5B).

### Breast tumors with altered (high and low) expression(s) of *MERIT40* and/or *WWOX* are associated with aneuploidy and poor prognosis

Excessive HR activity has been shown to induce aneuploidy, a hallmark of cancer [8]. We showed that WWOX inhibits MERIT40-induced HR activity, that *MERIT40* is overexpressed in breast tumors, and that this overexpression is a marker of poor prognosis in BC. These observations led us to examine the correlations between the expressions of *MERIT40* and *WWOX* genes, and, aneuploidy and prognosis in a cohort of breast tumors from the TCGA database.

**Fig. 6:**
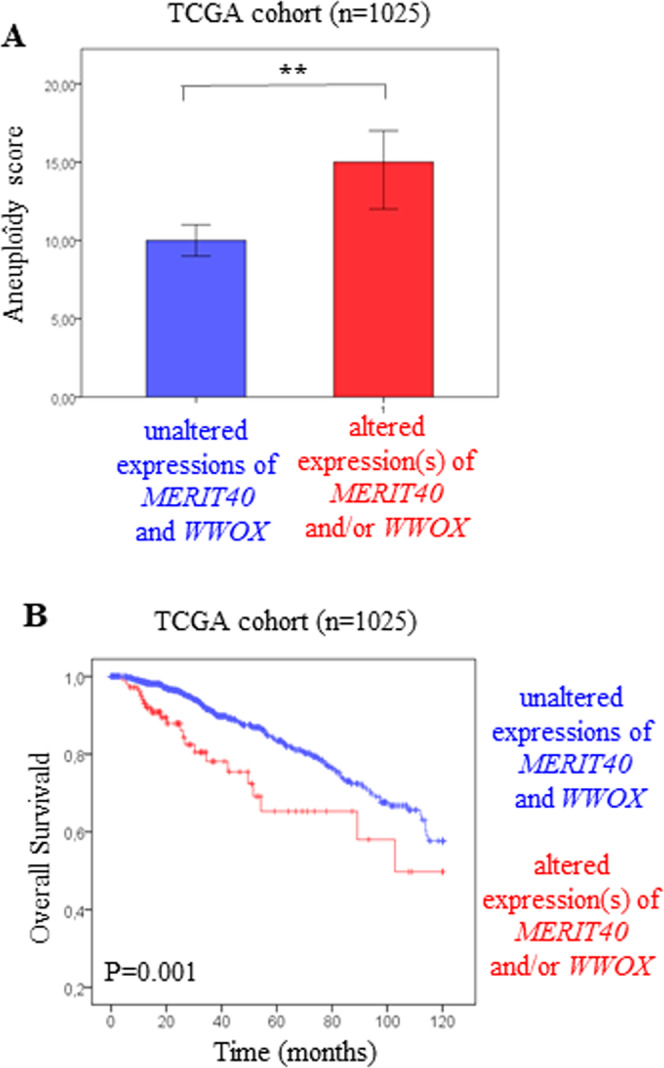
Breast tumors with altered expression(s) of *MERIT40* and/or *WWOX* are associated with aneuploidy and poor prognosis. **A** Levels of the aneuploidy score in tumors of the TCGA-breast cohort (*n* = 1025) presenting unaltered expressions of *MERIT40* and *WWOX* (blue bar) versus altered expression(s) of *MERIT40* and/or *WWOX* (red bar). **B** Kaplan–Meier curves showing the metastasis-free survival rates of patients with tumors presenting unaltered expressions of *MERIT40* and *WWOX* (blue line) versus altered expression(s) of *MERIT40* and/or *WWOX* (red line) in human breast tumors from the TCGA cohort (*n* = 1025).

We found that tumors with altered expression(s) of *MERIT40* and/or *WWOX* had higher aneuploidy scores than tumors with unaltered expressions of *MERIT40* and *WWOX* (*p* = 0.009, Mann–Whitney test, Fig. 6A), indicating the existence of less genomic instability in these last. In addition, the patients with the breast tumors with altered expression(s) of *WWOX* and/or *MERIT40*, were associated with a poor clinical outcome as compared with patients with unaltered tumors (*p* = 0.001, Fig. 6B).

These results suggest that altered expression of *MERIT40* or *WWOX*, would enhanced BC, at least in part, by increasing aneuploidy.

## Discussion

We demonstrated that MERIT40 stimulates HR activity (Fig. 2). This is in agreement with various studies and especially that of Jiang and al. showing a decrease of sister-chromatid exchange in *Merit40*^−/−^ MEFs after etoposide-induced DSBs, and a decrease in single strand DNA levels in *Merit40*^−/−^ splenocytes after IR [40]. Our results are also supported by Okamoto’s work showing that to induce DDR, MERIT40 must recruit Tankyrase, an activator of HR, to DSB induced by IR [18, 21]. Once located to DSBs, Tankyrase recruits various HR proteins such as CtIP and RAD51. Therefore, MERIT40 would stimulate HR, at least in part, by recruiting Tankyrase to DSBs. Nagy and al. have shown that Tankyrase is first recruited by MDC1 and then recruits BRCA1-A to DSBs [21]. Tankyrase and MERIT40 therefore seem to favor each other’s recruitment.

We showed that WWOX negatively modulates HR activity in Hela cells (Fig. 2). This result is in agreement with a recent work by Schrock and al. showing that WWOX also inhibits HR in the human glioblastoma cell line U87 and the human breast cell line MDA-MB-231 [28]. These authors further reported that WWOX inhibits HR in human breast cell lines by interacting with BRCA1, which leads to the inhibition of the formation of the two complexes indispensable for resection and therefore for HR: BRCA1-B and BRCA1-C [29]. However, Aqeilan and al. found that WWOX activates HR in the osteosarcoma cells U2OS, by interacting with ATM and stimulating its kinase activity [26]. The effect of WWOX on HR seems therefore to depend on the cell type. We revealed a novel molecular mechanism by which WWOX inhibits HR in BC. We identified MERIT40 as a new molecular partner of WWOX and showed that the WWOX-MERIT40 interaction inhibits the ability of MERIT40 to interact with Tankyrase. We detected the endogenous complex WWOX-MERIT40 in the breast cell line MCF7, suggesting that this novel mechanism is functional in breast cells. Tankyrase also plays a positive role in NHEJ by stabilizing the kinase DNA-PK [41]. It is conceivable that the Tankyrase-MERIT40 interaction has a positive role in HR and that Tankyrase, in the absence of MERIT40, favors NHEJ. Inhibition of the Tankyrase-MERIT40 interaction by WWOX would therefore suppress the stimulating role of this complex during HR but facilitate the role of Tankyrase during NHEJ.

MERIT40 enhances HR by recruiting Tankyrase to DSBs [[18, 21], and our results]. However, it is well established that MERIT40 is also a member of the nuclear complex BRCA1-A, that MERIT40 is indispensable to the formation of this complex, and that BRCA1-A eliminates excessive HR by limiting the extend of DNA end resection [7, 20]. To do this, BRCA1-A sequesters BRCA1 in DNA regions away from the DSBs [10, 42]. We found that WWOX promotes the formation of BRCA1-A (unpublished data). This finding explains, at least in part, why ectopic expression of WWOX increased MERIT40 nuclear foci (Fig. 3). The interaction of WWOX with MERIT40 would both enhance the positive effect of MERIT40 on the formation of the nuclear complex BRCA1-A, and prevent the MERIT40-Tankyrase interaction, thus inhibiting HR.

HR must be regulated very precisely because low or excessive activity of this DNA repair mechanism induces genome instability, such as aneuploidy, which can cause cancers [5–10]. Indeed, it has been reported that inhibition of expression of BRCA1 inhibits HR activity and induces evidence of aneuploidy [8, 10]. Hu and collaborators demonstrated that ectopic expression of BRCA1-D5, a BRCA1 mutant, promotes aneuploidy by inducing excessive activity of HR [8]. Moreover, an overexpression of *BRCA1* associated with aneuploidy and poor prognosis was observed in BC [10]. Therefore, our results showing that *MERIT40* is overexpressed in BC (Fig. S2), that *MERIT40* overexpression overstimulates HR activity (Fig. 2) and is associated with a poor prognosis (Fig. 5A), and that altered *MERIT40* expression is associated to aneuploidy (Fig. 6) suggest that MERIT40 overexpression, as that of BRCA1, plays an important role in BC development by overstimulating HR. A overexpression of *MERIT40*, and a SNP (rs8170) in the *MERIT40* gene associated with a significant cancer risk were found in epithelial ovarian cancer, suggesting that *MERIT40* could be involved in the development of different cancers [43]. Therefore, WWOX, through its interaction with MERIT40, could inhibit excessive HR activity induces by overexpression of MERIT40 by preventing the MERIT40-Tankyrase association, thereby opposing the MERIT40-dependent stimulation of BC.

We found that altered expression(s) of *MERIT40* and/or *WWOX* are associated to a high score of aneuploidy (Fig. 6A), indicating that these two genes have to be precisely regulated to avoid genome alterations. Inhibition of *WWOX* expression enhances, and its overexpression inhibits, HR activity [28]. We showed that overexpression of MERIT40 enhances HR (Fig. 2A), it is therefore likely that its under expression inhibits HR activity. As low and high activities of HR can induce aneuploidy [8], these observations lead us to suppose that the deregulation of these two genes would promote aneuploidy by affecting HR activity. Further analysis have to be perform to validate these hypotheses.

## Materials and methods

### Cell culture

Human embryonic kidney 293 (HEK293) cells, and human uterine carcinoma cell Hela were maintained in Dulbecco’s modified Eagle’s medium supplemented with 10% fetal bovine serum and antibiotics (penicillin 50 mg/ml, streptomycin 50 mg/ml and neomycin 100 mg/ml). Cell lines were grown at 37 °C in a humidified atmosphere of 5% (v/v) CO2 in air.

### Antibodies

Antibodies used: anti-WWOX (already described, [31]), anti-WWOX (Santa Cruz Biotechnology, Santa Cruz, CA, USA, sc-374449), anti-MERIT40 (Cell Signaling Technology, Danvers, MA, USA, 06/2017), anti-GAPDH (Santa Cruz, sc-47724), anti-Flag (Sigma, St Louis, MO, USA, F3165), anti-HA (Covance, VA, USA, MMS-101P), anti-Myc (Clontech, Palo Alto, CA, USA, 631206), anti-His (Santa cruz, sc-8036), alexa fluor 568-conjugated goat antibody anti-mouse (Invitrogene, A11031), alexa fluor 488-conjugated goat antibody anti-rabbit (Invitrogen, Carlsbad, CA, USA, A11034), horseradish peroxidase-conjugated goat antibody anti-rabbit (Jackson ImmunoResearch Laboratories, West Grove, PA, 111-035-003), and horseradish peroxidase-conjugated goat antibody anti-mouse (Jackson immunoresearch, 115-035-062).

### Plasmids

HA-WWOX, His-WWOX, His-WW1-WW2, His-WW2-SDR, Myc-WWOX, and Myc-WWOX_Y33R_ have already been described [31, 32]. QuickChange kit was used to obtain HA-WWOXY33R by site-directed mutagenesis (Stratagene, La Jolla, CA, USA). HA-MERIT40 and Flag-MERIT40 were constructed by inserting the appropriate cDNA, obtained by PCR using Myc-Flag-MERIT40 plasmid as template, into pCMV-HA (BD Bioscience Clontech, Palo Alto, CA, K6003-1) and p3xFLAG-CMV-7.1 vectors (Sigma, E4026) respectively. HA-ABRAXAS was constructed by inserting the appropriate cDNA, obtained by PCR using pOZ-N-FH Abraxas plasmid (Addgene, Cambridge, MA, USA, 27495) as template, into pCMV-HA vector. Myc-Flag-MERIT40 (RC202644), Myc-Flag-BRCC36 (RC224289), and Myc-Flag-RAP80 (RC202823) were purchased from OriGene (OriGene Technologies, Rockville, Md, USA). Flag-BRCA1 (52504) was purchased from Addgene, and Flag-BRE (HG16514-NF) from Clinisciences (Montrouge, France).

### Yeast two-hybrid cloning and analysis

The yeast two-hybrid has been performed in collaboration with Hybrigenics. The methods have been described in detail elsewhere [31].

### Co-immnunoprecipitation and western blot

Western blotting and co-immunoprecipitation methods have previously been described [31]. For endogenous WWOX-MERIT40 complex, cell extracts containing 800 µg of total proteins were subjected to direct immunoprecipitation with the anti-WWOX antibody produced by Eurogentec [31].

### Visualization of foci

Cells grown on coverslips were washed in PBS, permeabilized with EB buffer (50 mM NaCl, 5 mM MgCl, 300 mM sucrose, 0.5% Triton X-100, 10 mM Tris pH 6,8) for 5 min on ice, and fixated in EB buffer containing 4% paraformaldehyde for 15 min at room temperature. The coverslips were incubated with indicated primary antibodies over night at 4 °C. The cells were then washed with PBS and incubated with appropriate secondary antibodies (alexa fluor 568-conjugated goat antibody anti-mouse and alexa fluor 488-conjugated goat antibody anti-rabbit) for 2 h at room temperature. After washing with PBS, DNA was stained with 1 µg/ml DAPI. The coverslips were mounted with Prolong Diamond antifade Mountant (Invitrogen). Images were taken using a fluorescence microscope (Eclipse Ti-S nikon, Melville, NY). Z-dimension series of image were taken every 0.2 µm for each images of nuclei and were deconvoluted. All acquisition, analyses, and quantification were performed using the NIS-Elements AR (Nikon) and the ImageJ imaging software.

### siRNA experiments

siRNA control (Catalog no.: 1027281) and siRNA WWOX (Catalog no.: SI02777775) were purchased from Qiagen (Valencia, CA, USA). Cells were transfectected with the siRNA by using HiPerfect transfection Reagent (Qiagen) according to the manufacturer’s instructions.

### BC patients, tumor samples, and mammary cell line samples

Samples of 499 primary unilateral invasive breast tumors excised from women managed at Curie Institute-René Huguenin Hospital (Saint-Cloud, France) from 1978 to 2008 have been analysed. The malignancy of infiltrating carcinomas was scored according to Scarff–Bloom–Richardson’s (SBR) histoprognostic system. Hormone receptors (estrogen receptor α (ERα), progesterone receptor (PR), ER/PR) and human epidermal growth factor receptor 2 (ERBB2) status were determined at the protein level by using biochemical methods (dextran-coated charcoal method, enzyme immunoassay or immunohistochemistry) and confirmed by real-time quantitative RT-PCR assays. The population was classified into four subtypes according to ER/PR and ERBB2 status, as follows: ER/PR+ ERBB2+ (*n* = 53) ER/PR+ ERBB2− (*n* = 285), ER/PR− ERBB2+ (*n* = 65) and ER/PR− ERBB2− (triple-negative tumors) (*n* = 96). The breast cell lines were obtained from the American Type Culture Collection (ATCC, Manassas, VA, USA) or from the German Resource Centre for Biological Material (DSMZ, Braunschweig, Germany) and were cultured in the conditions recommended by suppliers. The triple-negative series consisted of 96 triple-negative breast tumors from patients treated by first-line surgery at the Institut Curie. All tumors were negative for ER, PR (<1% nuclear staining by immunohistochemistry (IHC)), and ERBB2 (<2+ by IHC or non-amplified by fluorescent in situ hybridization). Tumor RNA extracted from frozen tissue were obtained from the Institut Curie Biological Resource Center.

### Real-time quantitative PCR

The theoretical basis, RNA extraction, cDNA synthesis, design of primers and qRT-PCR conditions have been previously described in detail [44]. The nucleotide sequences of the primers used for real-time RT-PCR amplification were as follows: *WWOX*-U: 5′-CTG GGT TTA CTA CGC CAA TCA CA-3′, *WWOX*-L: 5′-GCA AAT CTC CTG CCA CTC GTT-3′, *MERIT40*-U: 5′-GAT GGG AGC CTCA ACA CTT CAG-3′, *MERIT40*-L: 5′-ACA GGT CCA GGC AGA TAA TCA CTT-3′. mRNA expression values for tumor samples were normalized such that the median value of the breast tumors cohorts (Fig. 5 and Fig. 6) or normal breast tissue (Fig. S2) was equal to 1.

### Statistical analysis

The *P* values less than 0.05 were considered to be statistically significant (*p < 0.05; **p < 0.01; ***p < 0.001). Comparisons were performed using two-sided unpaired Student *t* (Figs. 2 and 6) and Wilcoxon signed rank (Fig. 4) tests. Relationships between *MERIT40* mRNA expression and classical clinical biological parameters were assessed by using the Chi-squared and the non-parametric Mann–Withney tests (Table 3). To visualize the efficacy of a molecular marker (*MERIT40* mRNA level) to discriminate between two populations (patients who developed/did not develop metastases) in the absence of an arbitrary cutoff value, data were summarized in a ROC (receiver operating characteristic) curve [45]. The area under the ROC curve (AUC) was calculated as a single measure to discriminate efficacy. This single measure was used to separate *MERIT40* mRNA expression in two groups (Fig. 5 and Table 3). Metastasis-free survival (MFS) was determined as the interval between initial diagnosis and detection of the first metastasis. Survival distributions were estimated by the Kaplan–Meier method, and the significance of differences between survival rates were ascertained with the log-rank test (Fig. 5). In figure 9, the hierarchical clustering was performed with GeneANOVA software [46]. The correlation between *MERIT40* mRNA expression and LST number, and the significance differences between the three groups in terms of LST status were ascertained with the non-parametric Mann Withney and Chi-squared tests respectively. The multivariate Cox proportional hazards regression model was used to assess the independent contribution of each variable to metastasis-free survival. The results are presented as hazard ratios (HR) and 95% confidence intervals (95% CI).

## Supplementary information

S1 and S2

## Data Availability

The data sets used in this study can be obtained from TCGA.
