# Supplementary material for: WWOX binds MERIT40 and modulates its function in homologous recombination, implications in breast cancer
Source: Cancer Gene Ther. 2023 May 29;30(8):1144–55. doi: 10.1038/s41417-023-00626-x (PMC10425285; doi:10.1038/s41417-023-00626-x)
Supplement: Supplementary file 1 — S1 and S2 [file 41417_2023_626_MOESM1_ESM.ppt]

## Slide 1
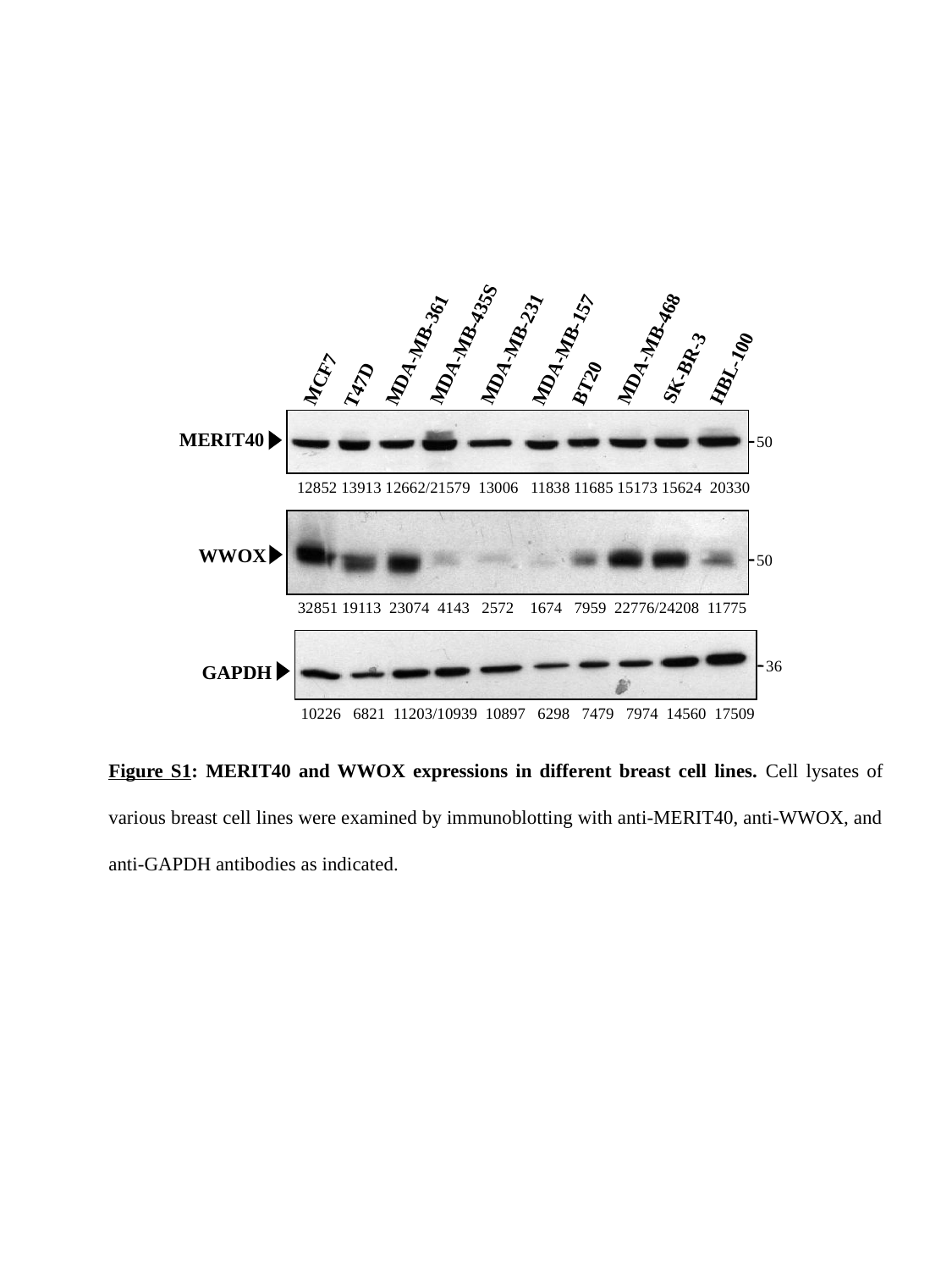

MDA-MB-435S
MDA-MB-231
MDA-MB-468
MDA-MB-361
MDA-MB-157
SK-BR-3
HBL-100
MCF7
BT20
T47D
MERIT40
50
12852 13913 12662/21579 13006 11838 11685 15173 15624 20330
WWOX
50
32851 19113 23074 4143 2572 1674 7959 22776/24208 11775
36
GAPDH
10226 6821 11203/10939 10897 6298 7479 7974 14560 17509
Figure S1: MERIT40 and WWOX expressions in different breast cell lines. Cell lysates of various breast cell lines were examined by immunoblotting with anti-MERIT40, anti-WWOX, and anti-GAPDH antibodies as indicated.

## Slide 2
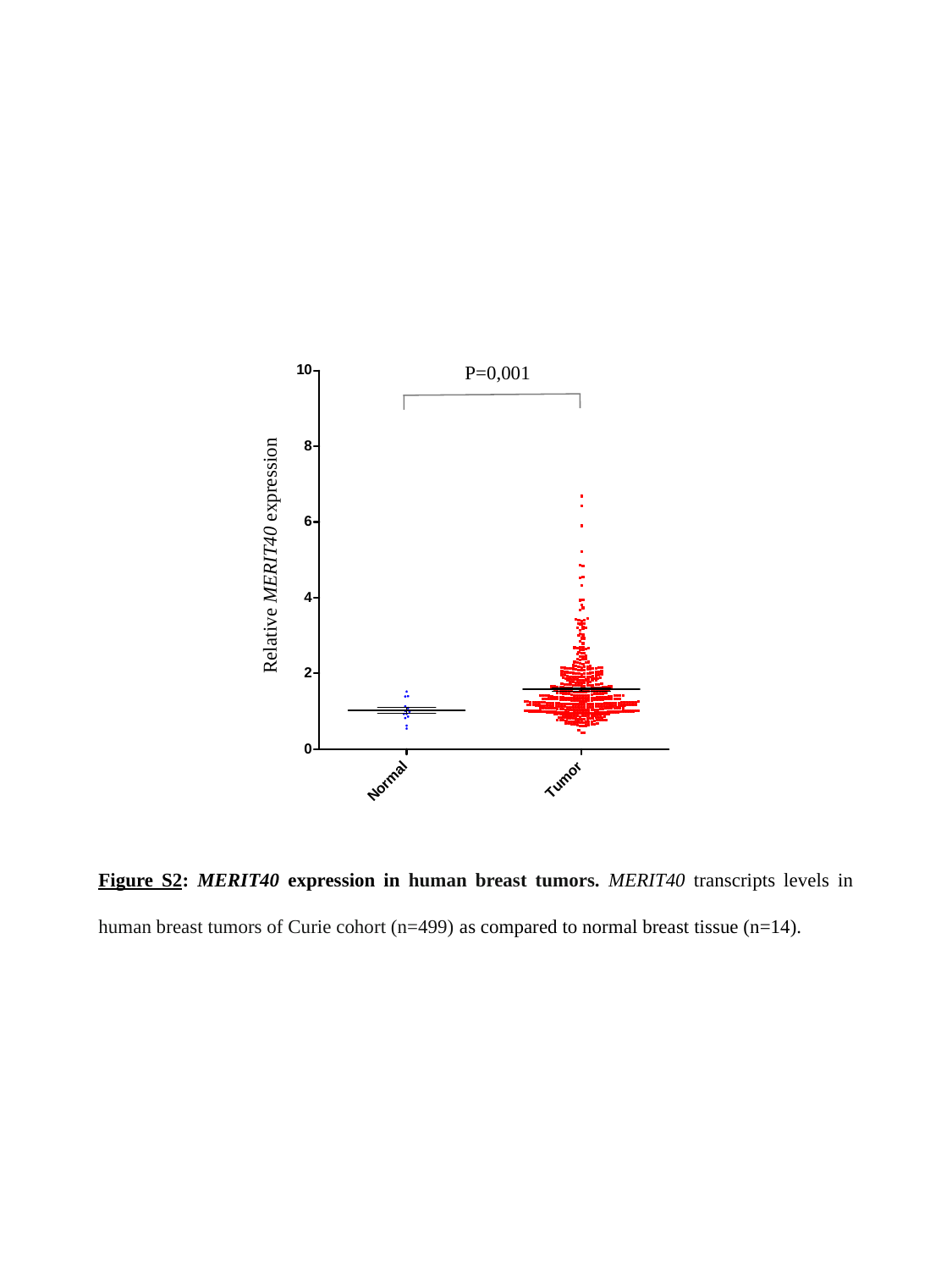

P=0,001
Relative MERIT40 expression
Figure S2: MERIT40 expression in human breast tumors. MERIT40 transcripts levels in human breast tumors of Curie cohort (n=499) as compared to normal breast tissue (n=14).
